# Childhood maltreatment and amygdala connectivity in methamphetamine dependence: a pilot study

**DOI:** 10.1002/brb3.289

**Published:** 2014-09-29

**Authors:** Andy C Dean, Milky Kohno, Gerhard Hellemann, Edythe D London

**Affiliations:** 1 Department of Psychiatry and Biobehavioral Sciences and Semel Institute, David Geffen School of Medicine, University of California Los Angeles760 Westwood Plaza, 90024, Los Angeles, California, USA; 2 Brain Research Institute, University of California Los AngelesLos Angeles, California, 90095, USA; 3 Department of Molecular and Medical Pharmacology, University of California Los AngelesLos Angeles, California, 90095, USA

**Keywords:** Amygdala, brain imaging, childhood, connectivity, drug, fMRI, maltreatment, methamphetamine, substance abuse, trauma

## Abstract

**Introduction:**

Childhood maltreatment, a well-known risk factor for the development of substance abuse disorders, is associated with functional and structural abnormalities in the adult brain, particularly in the limbic system. However, almost no research has examined the relationship between childhood maltreatment and brain function in individuals with drug abuse disorders.

**Methods:**

We conducted a pilot study of the relationship between childhood maltreatment (evaluated with the Childhood Trauma Questionnaire; Bernstein and Fink 1998) and resting-state functional connectivity of the amygdala (bilateral region of interest) with functional magnetic resonance imaging in 15 abstinent, methamphetamine-dependent research participants. Within regions that showed connectivity with the amygdala as a function of maltreatment, we also evaluated whether amygdala connectivity was associated positively with negative affect and negatively with healthy emotional processing.

**Results:**

The results indicated that childhood maltreatment was positively associated with resting-state connectivity between the amygdala and right hippocampus, right parahippocampal gyrus, right inferior temporal gyrus, right orbitofrontal cortex, cerebellum, and brainstem. Furthermore, connectivity between the amygdala and hippocampus was positively related to measures of depression, trait anxiety, and emotion dysregulation, and negatively related to self-compassion and dispositional mindfulness.

**Conclusions:**

These findings suggest that childhood maltreatment may contribute to increased limbic connectivity and maladaptive emotional processing in methamphetamine-dependent adults, and that healthy emotion regulation strategies may serve as a therapeutic target to ameliorate the associated behavioral phenotype. Childhood maltreatment warrants further investigation as a potentially important etiological factor in the neurobiology and treatment of substance use disorders.

## Introduction

Childhood maltreatment is a risk factor for the subsequent abuse of drugs. High rates of childhood maltreatment have been reported by adults who abuse methamphetamine (Meade et al. 2012), heroin (Wang et al. 2010), cocaine (Afifi et al. 2012), alcohol (Fenton et al. 2013), and nicotine (Mingione et al. 2012). Childhood maltreatment is also associated with an earlier onset of drug abuse (Messina et al. 2008), greater likelihood of progression to injection drug use (Debeck et al. 2013), more severe negative consequences from substance abuse (Asberg and Renk 2012), and greater likelihood of relapse following abstinence (Heffner et al. 2011). Notably, childhood maltreatment is related to drug abuse in adulthood whether or not the maltreatment leads to a subsequent diagnosis of post-traumatic stress disorder (PTSD; Fetzner et al. 2011).

Independent of drug abuse, childhood maltreatment has been associated with structural and functional abnormalities in the brain. Adults with a history of maltreatment exhibit volumetric deficits in the prefrontal cortex (PFC), hippocampus, corpus callosum (particularly middle and posterior regions), and cerebellum (see Hart and Rubia 2012). Despite the role of the amygdala in regulating responses to stressful and emotional stimuli, results from studies examining the relationship between amygdala volume and a history of maltreatment have been inconclusive, with no significant relationship in a meta-analysis (Woon and Hedges 2008). Studies of emotional reactivity, however, consistently show that the amygdala is hyper-responsive to fearful and threatening images, particularly faces, in adults with a history of maltreatment (e.g., Hart and Rubia 2012; Dannlowski et al. 2013; McCrory et al. 2013). Furthermore, individuals who report maltreatment exhibit abnormalities in activation of the dorsolateral prefrontal cortex (DLPFC) and anterior cingulate cortex (ACC) during tests of working memory and inhibitory control (Hart and Rubia 2012). Resting-state functional connectivity (RSFC) has also been examined in individuals with a history of maltreatment; however, the results have been mixed. Two studies found less connectivity between limbic and cortical regions (e.g., amygdala, hippocampus, insula, precuneus) in individuals with histories of maltreatment relative to those reporting no maltreatment (Hart and Rubia 2012; van der Werff et al. 2013; Wang et al. 2014), while another study (Philip et al. 2013) found that maltreated individuals had less RSFC in midline cortical regions than control subjects, but greater connectivity between the amygdala and medial prefrontal cortex (PFC).

Childhood maltreatment places individuals at greater risk for developing other various forms of psychological distress, including depression, anxiety, emotional dysregulation, impulsivity, and suicidality (see Gilbert et al. 2009). Survivors of childhood maltreatment also have increased odds of developing a broad range of psychiatric conditions (Sugaya et al. 2012). Despite the need for maltreated individuals to cope with the emotional distress they experience, they may fail to utilize adaptive coping strategies to manage their affect. For example, childhood maltreatment has been negatively related to the tendency to treat oneself with compassion (Tanaka et al. 2011). This deficit in self-compassion may reflect the manner in which maltreatment during the formative years can result in a negative view of oneself and the standards by which he/she should be treated.

Despite the associations between childhood maltreatment, substance abuse, and brain function, there has been a paucity of research on the potential links between these entities. To address this gap in the literature, the goal of this pilot study was to examine the relationship between childhood maltreatment and resting-state connectivity of the amygdala in a sample of fifteen abstinent methamphetamine-dependent adults with no history of PTSD. Inconsistencies and omissions in the literature complicated the development of firm hypotheses. Nonetheless, given evidence that methamphetamine-dependent research participants have elevated amygdala glucose metabolism that is positively associated with trait anxiety (London et al. 2004), and the aforementioned hyperactivity of the amygdala to emotional stimuli in maltreated individuals, it was hypothesized that maltreatment would be positively associated with connectivity between the amygdala and other limbic and cortical regions in methamphetamine-dependent participants. Next, within the regions that showed significant connectivity with the amygdala as a function of maltreatment, we evaluated whether connectivity was linearly related to measures of current negative affect (i.e., depression, anxiety, emotional dysregulation) and adaptive emotional processing (i.e., self-compassion, trait mindfulness). The primary hypotheses were that connectivity in the regions associated with maltreatment would be positively related to negative affect and negatively related to adaptive emotional processing.

## Methods

Analyses were conducted on data from 15 methamphetamine-dependent volunteers who tested positive for methamphetamine in urinalyses at intake. Participants were recruited using Internet and local newspaper advertisements. After receiving a detailed description of the protocol, they provided written informed consent, following the guidelines of the UCLA Office for Protection of Research Subjects. Nine of these participants completed the study as inpatients at the UCLA General Clinical Research Center (GCRC), and six completed the study as outpatients after a change in funding to support inpatient studies. Some of the subjects participated in other studies of brain structure or function in methamphetamine dependence (Morales et al. 2012; Kohno et al. 2014), or a study of medication (i.e., modafinil) to improve cognitive control in methamphetamine-dependence (Ghahremani et al. 2011). If a participant was part of the medication study, all procedures in the current study were conducted prior to the administration of medication or placebo. Urinalyses confirmed abstinence on the fMRI scanning day, which followed 4–12 days (mean 7.5 ± 2.6) of abstinence from illicit drugs of abuse. All participants were fluent in English and were administered the Structured Clinical Interview for the DSM-IV (SCID) for Axis I diagnosis (First et al. 1995). *Exclusion criteria*, based on interview and laboratory tests, were: pregnancy, neurological disease (e.g., stroke, head trauma with loss of consciousness >30 min); frank structural brain abnormalities on MRI; systemic disease; cardiovascular disease; pulmonary disease; HIV infection (HIV1/HIV2 antibody screen); abnormal laboratory tests (hematocrit, plasma electrolytes, markers for hepatic and renal function); and use of medications that affect the central nervous system. Any current Axis I diagnosis other than nicotine dependence and methamphetamine dependence was exclusionary. Demographic information on the participants is presented in Table 1.

**Table 1:** Characteristics of the methamphetamine-dependent participants (*N *=* *15)

Age (years)	38.40 ± 2.30
Gender (# male)	8
Education (years)	12.93 ± 0.44
Days used alcohol last 30 days	2.07 ± 0.83
Days used marijuana last 30 days	1.69 ± 1.15
Tobacco use (# smokers)	13
Cigarette pack years (smokers only)	9.52 ± 3.04
Days used methamphetamine last 30 days	22.93 ± 6.57
Years of regular methamphetamine use	7.80 ± 4.89

Data reflect mean and standard deviation; Regular methamphetamine use = using at least 3 days per week, or twice weekly binges.

### Questionnaires

#### Childhood maltreatment

Childhood maltreatment was assessed with the Childhood Trauma Questionnaire (CTQ; Bernstein and Fink 1998), a 25-item retrospective self-report that assesses five primary domains: physical abuse, sexual abuse, emotional abuse, physical neglect, and emotional neglect. Items include, “I believe I was physically abused as a child” (physical abuse) and “People in my family felt close to each other” (reverse score, emotional neglect). Participants respond on a 5-point scale, ranging from “1”, indicating “never true” to “5”, denoting “very often true.” Research supports the reliability and validity of the CTQ (Bernstein and Fink 1998; Paivio and Cramer 2004). The total CTQ score, calculated as the sum across all items, was used as a measure of childhood maltreatment.

#### Negative affect

Symptoms of depression, trait anxiety, and difficulties with emotion regulation were self-reported on the Beck Depression Inventory (BDI, Beck and Beamesderfer 1974), the State-Trait Anxiety Inventory (STAI, Trait Scale, Spielberger and Gorsuch 1983), and the Difficulties in Emotion Regulation Scale (DERS, Gratz and Roemer 2004) respectively. The number of items on these scales ranges from 20 (Trait Anxiety) to 36 (DERS), and responses are obtained using a Likert-style format. All three scales have been well-validated and are commonly used in research on childhood maltreatment (e.g., Nanni et al. 2012; Dannlowski et al. 2013; Weiss et al. 2013).

#### Adaptive emotional processing

We measured the self-report of two adaptive emotion-processing strategies: the tendency to treat oneself with compassion, measured by the 26-item Self-Compassion Scale (SCS, Neff 2003), and dispositional mindfulness, measured by the 15-item Mindful Attention Awareness Scale (MAAS, Brown and Ryan 2003). Both scales use Likert-style items, with scores ranging from “almost never” to “almost always.” The SCS measures self-kindness (as opposed to self-judgment) during emotional pain and a feeling of common humanity when things go wrong, whereas the MAAS measures the tendency to pay attention and be aware of moment-to-moment experience (for information and validation, see Brown and Ryan 2003; Neff 2003).

#### fMRI resting-state scanning

Imaging was performed at 3 Tesla on a Siemens Magnetom Trio MRI system. For the resting-state scan, participants were presented with a black screen for 5-min and were asked to keep their eyes open. A set of 152 functional, T2*-weighted, echoplanar images (EPI) was acquired (slice thickness = 4 mm; 34 slices; repetition time (TR) = 2 sec; echo time (TE) = 30 ms; flip angle = 90°; matrix = 64 × 64; field of view = 200 mm). High-resolution, T2-weighted, matched-bandwidth and magnetization-prepared rapid-acquisition gradient echo (MPRAGE) scans were also acquired. The orientation for matched-bandwidth and EPI scans was oblique axial to maximize brain coverage and to optimize signal from ventromedial prefrontal regions.

#### Analysis of fMRI resting-state data (amygdala seed)

Image analysis was performed using the FMRIB Software Library (FSL) (5.0.2.1) (www.fmrib.ox.ac.uk/fsl). The image series from each participant was first realigned to compensate for small head movements (Jenkinson et al. 2002), and high-pass temporal filtering was applied (Gaussian-weighted least-squares straight-line fitting, with sigma = 33s). Data were spatially smoothed using a 5-mm FWHM Gaussian kernel, and skull-stripping was performed using the FSL Brain Extraction Tool. Regis-tration was conducted through a three-step procedure, whereby EPI images were first registered to the matched-bandwidth structural image, then to the high-resolution MPRAGE structural image, and finally into standard Montreal Neurological Institute space, using 12-parameter affine transformations. Registration of MPRAGE structural images to standard space was further refined using FNIRT nonlinear registration (Andersson et al. 2007). Statistical analyses were performed on data in native space using FMRIB's fMRI Expert Analysis Tool (FEAT). Data were further processed to include nuisance regressors reflecting the average signal generated by cerebrospinal fluid and two additional motion-related confounds that measure head displacements and volume-by-volume BOLD signal displacements: frame-wise displacement (FD) and the root mean squared change in BOLD signal (DVARS) (Power et al. 2011). For all analyses, age, gender, years of regular methamphetamine use and inpatient status (yes/no) were included as nuisance covariates (outpatients were recruited by virtue of their ability to abstain independently, while inpatients were not). A bilateral amygdala ROI was anatomically derived from the Harvard-Oxford subcortical atlas. The mean time series of the amygdala ROI was calculated by averaging the time series of all voxels within the ROI. A whole-brain, voxel-wise regression analysis was performed in which CTQ scores were regressed onto the mean amygdala time series. Statistical thresholds were set at a voxel height of *Z* > 2.3 and a cluster probability of *P *<* *0.05, corrected for multiple comparisons.

#### Post-hoc analyses of amygdala resting-state connectivity, negative affect, and adaptive emotion processing

As indicated below, childhood maltreatment was positively associated with connectivity of the amygdala with regions of the temporal lobe, frontal lobe, and cerebellum, with particular involvement of the right hippocampus (see Results section). Given the theoretical importance of amygdala-hippocampal connectivity to both childhood maltreatment and emotion regulation (Hart and Rubia 2012; Kirby et al. 2012), we tested whether connectivity between the amygdala and right hippocampus was associated positively with negative affect and negatively with adaptive emotional processing. Using the voxel-wise connectivity values (*β*-values) from the aforementioned whole-brain regression analysis (which assessed the temporal correlations between activity in the amygdala seed and in the whole brain), we derived the average connectivity strength between the right hippocampus and amygdala, using an anatomically-defined ROI of the hippocampus. This provided a single value (*β*-value) of the strength in connectivity between the amygdala and right hippocampus for each methamphetamine-dependent participant. Using linear regression, we then tested the relationship between these connectivity values and measures of current negative affect (i.e., depression, anxiety, emotional dysregulation) and measures of adaptive emotional processing (i.e., self-compassion, trait mindfulness), controlling for age, gender, years of regular methamphetamine use and inpatient status.

## Results

### CTQ and amygdala RSFC

Childhood Trauma Questionnaire scores for the methamphetamine-dependent subjects are provided in Table 2. Relative to age and gender-based normative data from a community sample (Scher et al. 2001), CTQ total scores were greater than 1.5 standard deviations above the normative mean (95th percentile), with particular elevations in the domains of sexual abuse and emotional abuse/neglect. The CTQ total scores were also significantly higher than those of healthy control subjects of comparable age and gender (*N *=* *39), who were studied in our laboratory (*P *=* *0.004; data not shown). However, according to cutoffs provided in the CTQ manual (Bernstein and Fink 1998) which categorizes the severity of maltreatment (i.e., none, low, moderate, severe), the mean performance of the methamphetamine-dependent participants placed in the “low” range, with the exception of sexual abuse, which was “moderate.” Variability was exhibited in every domain, however, with scores ranging from “none” to “severe” in every maltreatment category.

**Table 2:** Descriptive statistics for measures of negative affect, adaptive emotional processing, and childhood maltreatment

Measure	Mean ± SD	Range	Normative percentile
Beck Depression Inventory^a^	10.13 ± 6.06	1–22	68th %ile
Spielberger Trait Anxiety^b^	40.20 ± 11.70	24–68	63rd %ile
Difficulties in Emotion Regulation^c^	70.85 ± 15.58	48–97	75th %ile
Self-Compassion Scale^d^	3.43 ± 0.51	2.70–4.55	46th %ile
Mindful Attention Awareness Scale^e^	67.87 ± 12.11	54–89	53rd %ile
Childhood Trauma Questionnaire^f^			
Total score	45.53 ± 18.83	26–88	95th %ile
Emotional abuse	10.40 ± 4.73	5–19	91st %ile
Emotional neglect	11.00 ± 4.74	5–20	90th %ile
Physical abuse	8.00 ± 3.95	5–19	71st %ile
Physical neglect	7.27 ± 2.60	5–14	73rd %ile
Sexual abuse	8.87 ± 6.60	5–25	>99th %ile

Normative percentiles for the mean of each scale were derived from community samples using the following sources:

a (Seggar et al. 2002);

b (Spielberger and Gorsuch 1983);

c (Fox et al. 2007);

d (Werner et al. 2012);

e (Carlson and Brown 2005); and

f (Scher et al. 2001).

**Figure 1:**
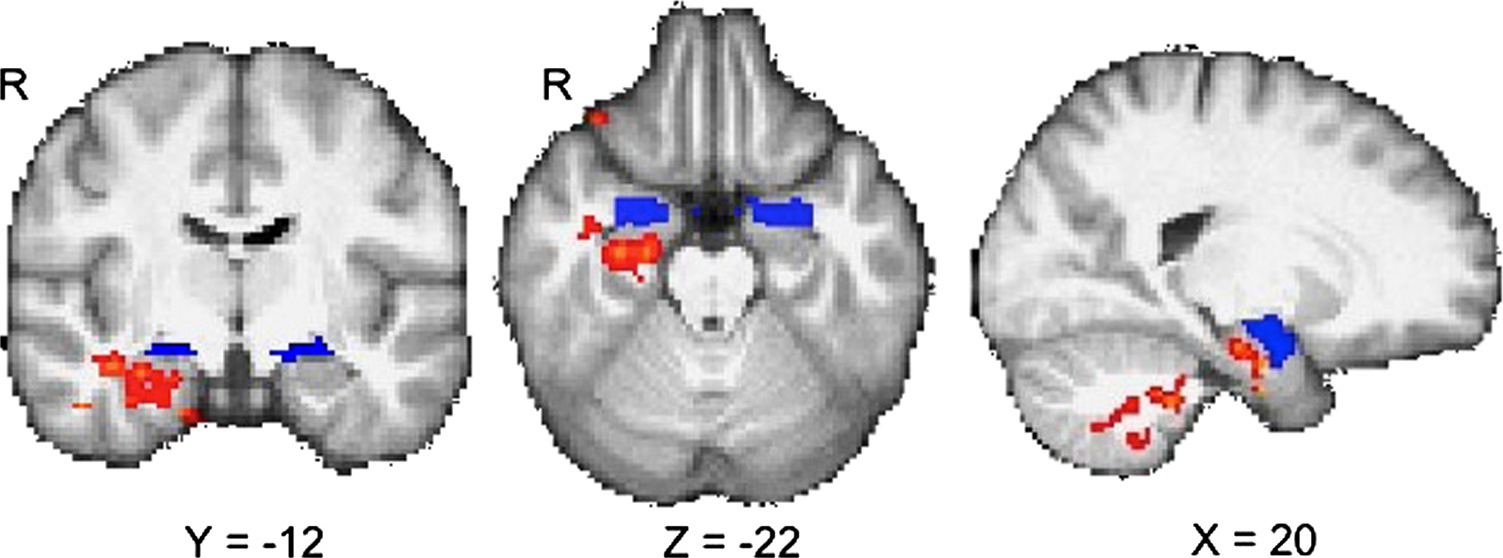
Relationship between amygdala resting-state connectivity and childhood maltreatment. Connectivity maps show a positive relationship between maltreatment and connectivity between the amygdala seed (shown in blue) and right hippocampus, right parahippocampal gyrus, right lateral orbitofrontal cortex, cerebellum, and brain stem in methamphetamine-dependent participants (*P *<* *0.05, whole-brain cluster-corrected). Results controlled for age, gender, years of regular methamphetamine use and inpatient versus outpatient status.

With respect to RSFC, CTQ total scores were positively related to connectivity strength between the amygdala and the right hippocampus, right parahippocampal gyrus, right inferior temporal gyrus, right orbitofrontal cortex (OFC), cerebellum, and brainstem (*P *<* *0.05, whole-brain, cluster corrected; see Fig.1). No areas of negative relationship were found between the CTQ and amygdala connectivity.

### Post-hoc analyses of amygdala-hippocampal RSFC, negative affect, and adaptive emotional processing

**Figure 2:**
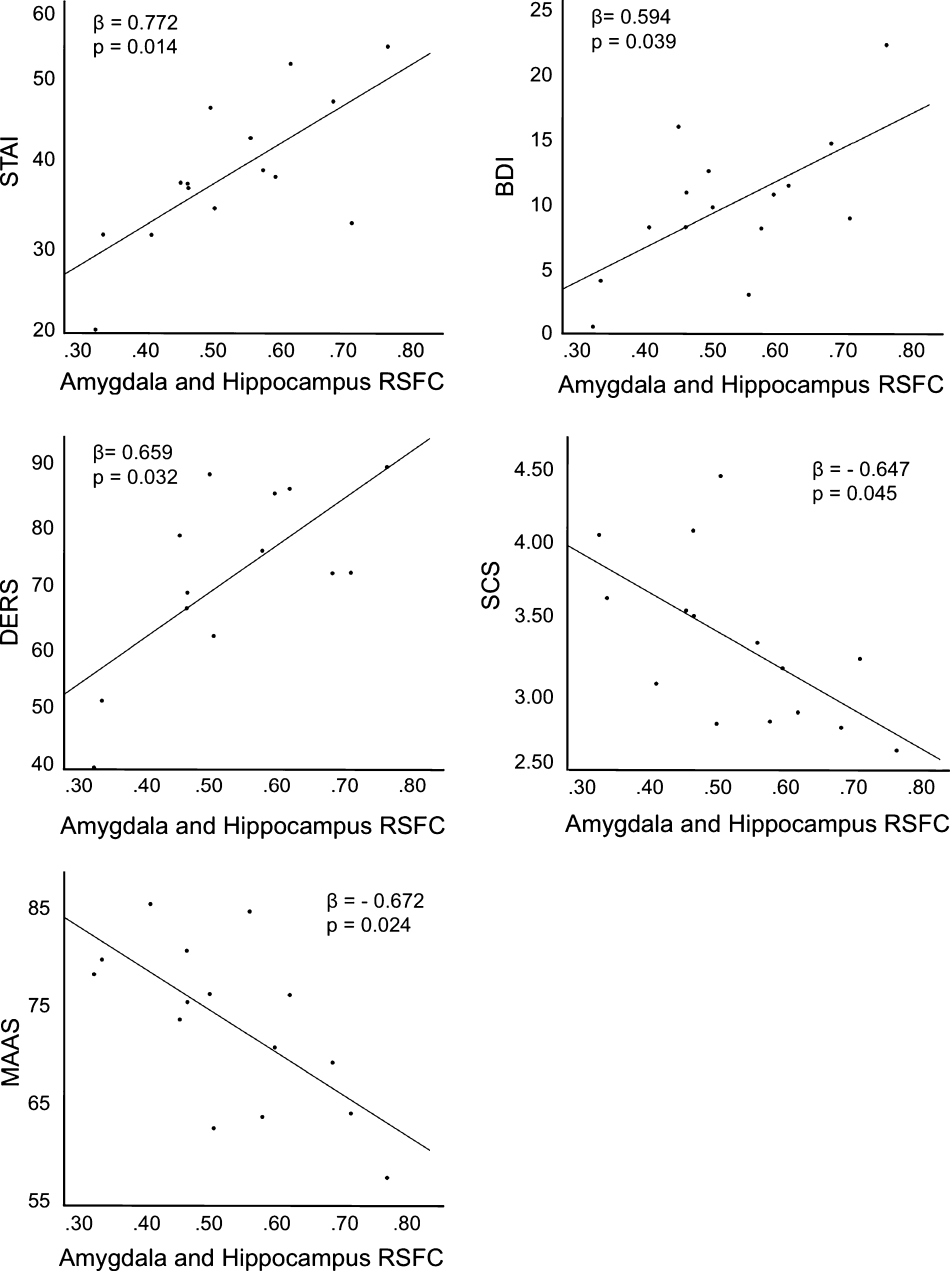
Scatterplots showing the relationship between connectivity between the amygdala and right hippocampus and measures of negative affect and adaptive emotional processing. RSFC, strength of resting-state functional connectivity (*β*-values); STAI, State-Trait Anxiety Inventory (Trait Scale); BDI, Beck Depression Inventory; DERS, Difficulties in Emotion Regulation Scale; SCS, Self-Compassion Scale; MAAS, Mindful Attention Awareness Scale. *β *= standardized regression coefficient. Analyses controlled for age, gender, years of regular methamphetamine use and inpatient versus outpatient status.

RSFC between amygdala and right hippocampus was positively related to depressive affect (BDI, *β * =  0.594, *P *=* *0.039), trait anxiety (STAI-T, *β * =  0.772, *P *=* *0.014) and emotion dysregulation (DERS, *β * =  0.659, *P *=* *0.032) (see Fig.2). In contrast, RSFC between amygdala and right hippocampus was negatively related to self-compassion (SCS, *β * =  −0.647, *P *=* *0.045) and mindful disposition (MAAS, *β * =  −0.672, *P *=* *0.024) (see Fig.2). In order to explore the possibility that maltreatment predicts negative affect and adaptive emotional processing through the mediation of amygdala-hippocampal RSFC, we first evaluated whether childhood maltreatment was significantly related to negative affect and adaptive emotional processing. Maltreatment was positively related to depression (*β* = 0.722, *P *=* *0.033) and trait anxiety (*β * =  0.714, *P *=* *0.011), and negatively related to self-compassion (*β * =  −0.609, *P *=* *0.050), while it was not significantly related to difficulties in emotion regulation or mindfulness (*P*'s > 0.05). Next, we used the Sobel test (Sobel 1982) to determine whether amygdala-hippocampal RSFC significantly mediates between maltreatment and depression, anxiety, or self-compassion (i.e., whether the indirect path was significant). RSFC was not a significant mediator in these analyses (*P*'s > 0.05), suggesting that amygdala-hippocampal RSFC is not the primary means through which maltreatment relates to these constructs.

## Discussion

In methamphetamine-dependent adults, childhood maltreatment was positively associated with RSFC between the amygdala and right hippocampus, right parahippocampal gyrus, right inferior temporal gyrus, right OFC, cerebellum and brainstem. Furthermore, connectivity between the amygdala and right hippocampus was positively related to measures of current depression, anxiety, and emotional dysregulation, but negatively related to self-compassion and dispositional mindfulness. These data suggest that maltreatment in childhood may result in enduring differences in brain function that are associated with negative affective states and reduced adaptive emotional processing in adulthood. Given the consistent evidence that maltreatment increases the risk of substance abuse (e.g., Afifi et al. 2012), these results raise the possibility that limbic connectivity and concomitant emotional distress foster the development of methamphetamine abuse as a means of drug-induced emotion regulation (cf. Baicy and London 2007).

The finding of increased limbic connectivity associated with maltreatment is similar to the results of a previous study, which found that maltreated individuals (without comorbid substance abuse) had greater connectivity between the amygdala and medial PFC than healthy control subjects (Philip et al. 2013). In that study, the CTQ also was used to assess maltreatment, with participants in the maltreatment group all having moderate to severe levels of maltreatment in at least one abuse domain (i.e., physical, emotional, or sexual). In contrast, two other studies found less connectivity between the amygdala and other limbic and cortical regions in maltreated individuals than in control subjects (van der Werff et al. 2013; Wang et al. 2014). These studies specifically examined individuals with a history of emotional neglect/abuse (van der Werff et al. 2013) and emotional or physical neglect (Wang et al. 2014), which contrasts with the more general assessment of childhood maltreatment in the study presented here. Furthermore, the participants in one of these studies had comorbid major depressive disorder (Wang et al. 2014), unlike participants in the present study. As such, discrepancies in results may be attributable to differences in the populations investigated or the manner in which maltreatment was operationalized.

The current study also differed from previous studies in the severity of maltreatment examined and methods used. Earlier studies of RSFC and maltreatment used a between-groups design to compare individuals with and without maltreatment. The severity of abuse in those groups varied from minimally present (more than once; van der Werff et al. 2013) to moderate-to-severe (Philip et al. 2013; Wang et al. 2014). Here, we tested for a linear relationship between maltreatment and amygdala RSFC, in a group in which maltreatment ranged from minimal to severe, but on average was in the “low” range. Such differences in methods and severity may contribute to discrepancies in results. For example, the linear relationship observed between maltreatment and RSFC here may be a function of the severity of maltreatment sampled. More extreme levels of maltreatment, for instance, could result in compensatory alterations in brain function, possibly reversing trends observed at lower levels. Additional studies with larger sample sizes, and a complete range of maltreatment severity, are needed to clarify these issues.

Despite some ambiguities in the literature, research has repeatedly associated maltreatment with abnormalities in the limbic system. Individuals with a history of maltreatment reliably demonstrate hyperactivity of the amygdala and other limbic areas during the presentation of stressful or fearful cues (see Hart and Rubia 2012). The hippocampus and amygdala are intimately involved in the response of the hypothalamic-pituitary-adrenal (HPA) axis to stressors, with evidence suggesting that chronic stress reorganizes these limbic circuits (Jankord and Herman 2008). The current results suggest further that maltreatment may contribute to limbic abnormalities in individuals who abuse methamphetamine, with resting-state connectivity between the amygdala and certain cortical regions increasing in relation to the severity of maltreatment experienced. Research has shown that amygdala-cortical connectivity is elevated in the aftermath of an acute stressor (van Marle et al. 2010), raising the possibility that chronic maltreatment may lead to chronically elevated amygdala-cortical connectivity in certain situations (e.g., at levels of maltreatment studied here).

Although speculative, it is possible that increased amygdala connectivity and the concomitant depression and anxiety observed may increase the vulnerability to developing a substance use disorder. For example, without considering maltreatment, methamphetamine-dependent subjects exhibit elevated glucose metabolism in the amygdala, and structural abnormalities in the amygdala and hippocampus (London et al. 2004; Thompson et al. 2004). Furthermore, in both humans and animals, trauma and early life stress prospectively predict the initiation and increase of alcohol and drug use (Gordon 2002), including the use of methamphetamine (Lewis et al. 2013). Psychologically, use of drugs to cope with resultant emotional distress has been proposed as one of the primary determinants of stress-induced vulnerability, both in terms of reducing negative affect (i.e., negative reinforcement) and increasing pleasure (i.e., positive reinforcement; Sinha 2001). It is unclear precisely how stress-induced neuroadaptations increase vulnerability to drug abuse, but studies support the view that alterations in the HPA axis and chronic glucocorticoid release may “sensitize” the mesolimbic dopamine system such that drugs become more rewarding, homeostatic mechanisms are dysregulated, and self-control is attenuated (see Sinha 2001).

Although previous neuroimaging studies of maltreated individuals have related brain activity and/or structure with negative mood states, this is the first to demonstrate that brain connectivity is associated with reduced healthy coping strategies. Specifically, while the strength of connectivity between the amygdala and right hippocampus was positively related to maltreatment, it was negatively associated with trait mindfulness and the tendency to treat oneself with compassion. Although these reductions in mindfulness and self-compassion are presumably a byproduct of development in an abusive context, training in self-compassion and mindfulness strategies may serve as a therapeutic approach to counteract the untoward effects of maltreatment on brain responsivity and emotion regulation. In this regard, a burgeoning research base supports the use of self-compassion and mindfulness techniques to ameliorate multiple forms of psychiatric distress (Neff and Germer 2012; Khoury et al. 2013), including addictive disorders (Brewer et al. 2011; Witkiewitz et al. 2012).

Of the different types of maltreatment evaluated by the CTQ in the methamphetamine-dependent participants, sexual abuse was the highest and averaged in the “moderate” range. Because sexual abuse tends to be disproportionately represented among female participants (Finkelhor et al. 1990), we evaluated whether gender differences affected CTQ scores in the sample studied here. No gender differences were observed on any scale or subscale (*P*'s > 0.20); in fact, male subjects reported just slightly more sexual abuse than females (9.4 ± 6.3 vs. 8.3 ± 7.4 respectively). In addition, there was no indication of a significant interaction between overall maltreatment and gender on amygdala-hippocampal connectivity (*P *=* *0.89). Future studies with larger sample sizes are required to determine if these preliminary observations reflect sample-specific results or generalizable patterns in methamphetamine-dependent individuals.

This study has some limitations. In particular, the sample size was small, possibly limiting generalizability of the findings to methamphetamine-dependent individuals in the community, including those with psychiatric comorbidities (we excluded these subjects; see Salo et al. 2011) or those who have experienced severe maltreatment (few subjects in this category were included here). Small sample size also afforded limited power for statistical analyses, particularly as it relates to testing the mediation of RSFC on the maltreatment-emotion processing relationship, and potential interactions between gender and trauma on RSFC. As such, null findings for these analyses may reflect insufficient power rather than the absence of relationships. Furthermore, the behavioral results would not survive Bonferroni correction for multiple statistical comparisons, underscoring the need to test these hypotheses with a larger sample size to confirm the findings. Finally, although the analyses controlled for years of regular methamphetamine use (a measure that was not correlated with maltreatment), it is unclear to what extent the maltreatment and RSFC relationships noted are specific to some aspect(s) of methamphetamine dependence. Use of a study design that includes a control group with comparable maltreatment without a history of substance abuse would be preferable for disentangling methamphetamine effects from those of maltreatment. Despite these limitations, this study provides preliminary evidence that childhood maltreatment contributes to limbic abnormalities and negative affect and emotional processing in methamphetamine-dependent adults. Further studies are warranted to help understand the neurobiology of limbic dysregulation in stimulant abuse with the goal of reducing distress and improving treatment outcomes in this population.

## References

[b1] Afifi TO, Henriksen CA, Asmundson GJ, Sareen J (2012). Childhood maltreatment and substance use disorders among men and women in a nationally representative sample. Can. J. Psychiatry.

[b2] Andersson J, Jenkinson M, Smith S (2007).

[b3] Asberg K, Renk K (2012). Substance use coping as a mediator of the relationship between trauma symptoms and substance use consequences among incarcerated females with childhood sexual abuse histories. Subst. Use Misuse.

[b4] Baicy K, London ED (2007). Corticolimbic dysregulation and chronic methamphetamine abuse. Addiction.

[b5] Beck AT, Beamesderfer A (1974). Assessment of depression: the depression inventory. Psychol. Meas. Psychopharmacol.

[b6] Bernstein DP, Fink L (1998). Childhood Trauma Questionnaire: a retrospective self-report manual.

[b7] Brewer JA, Mallik S, Babuscio TA, Nich C, Johnson HE, Deleone CM (2011). Mindfulness training for smoking cessation: results from a randomized controlled trial. Drug Alcohol Depend.

[b8] Brown KW, Ryan RM (2003). The benefits of being present: mindfulness and its role in psychological well-being. J. Pers. Soc. Psychol.

[b9] Carlson LE, Brown KW (2005). Validation of the Mindful Attention Awareness Scale in a cancer population. J. Psychosom. Res.

[b10] Dannlowski U, Kugel H, Huber F, Stuhrmann A, Redlich R, Grotegerd D (2013). Childhood maltreatment is associated with an automatic negative emotion processing bias in the amygdala. Hum. Brain Mapp.

[b11] Debeck K, Kerr T, Marshall BD, Simo A, Montaner J, Wood E (2013). Risk factors for progression to regular injection drug use among street-involved youth in a Canadian setting. Drug Alcohol Depend.

[b12] Fenton MC, Geier T, Keyes K, Skodol AE, Grant BF, Hasin DS (2013). Combined role of childhood maltreatment, family history, and gender in the risk for alcohol dependence. Psychol. Med.

[b13] Fetzner MG, McMillan KA, Sareen J, Asmundson GJ (2011). What is the association between traumatic life events and alcohol abuse/dependence in people with and without PTSD? Findings from a nationally representative sample. Depress. Anxiety.

[b14] Finkelhor D, Hotaling G, Lewis IA, Smith C (1990). Sexual abuse in a national survey of adult men and women: prevalence, characteristics, and risk factors. Child Abuse Negl.

[b15] First MB, Spitzer RL, Gibbon M, Williams JBW (1995). The structured clinical interview for DSM-IV axis I disorders (SCID-IP).

[b16] Fox HC, Axelrod SR, Paliwal P, Sleeper J, Sinha R (2007). Difficulties in emotion regulation and impulse control during cocaine abstinence. Drug Alcohol Depend.

[b17] Ghahremani DG, Tabibnia G, Monterosso J, Hellemann G, Poldrack RA, London ED (2011). Effect of modafinil on learning and task-related brain activity in methamphetamine-dependent and healthy individuals. Neuropsychopharmacology.

[b18] Gilbert R, Widom CS, Browne K, Fergusson D, Webb E, Janson S (2009). Burden and consequences of child maltreatment in high-income countries. Lancet.

[b19] Gordon HW (2002). Early environmental stress and biological vulnerability to drug abuse. Psychoneuroendocrinology.

[b20] Gratz KL, Roemer L (2004). Multidimensional assessment of emotion regulation and dysregulation: development, factor structure, and initial validation of the Difficulties in Emotion Regulation Scale. J. Psychopathol. Behav. Assess.

[b21] Hart H, Rubia K (2012). Neuroimaging of child abuse: a critical review. Front. Hum. Neurosci.

[b22] Heffner JL, Blom TJ, Anthenelli RM (2011). Gender differences in trauma history and symptoms as predictors of relapse to alcohol and drug use. Am. J. Addict.

[b23] Jankord R, Herman JP (2008). Limbic regulation of hypothalamo-pituitary-adrenocortical function during acute and chronic stress. Ann. N. Y. Acad. Sci.

[b24] Jenkinson M, Bannister P, Brady M, Smith S (2002). Improved optimization for the robust and accurate linear registration and motion correction of brain images. Neuroimage.

[b25] Khoury B, Lecomte T, Fortin G, Masse M, Therien P, Bouchard V (2013). Mindfulness-based therapy: a comprehensive meta-analysis. Clin. Psychol. Rev.

[b26] Kirby ED, Friedman AR, Covarrubias D, Ying C, Sun WG, Goosens KA (2012). Basolateral amygdala regulation of adult hippocampal neurogenesis and fear-related activation of newborn neurons. Mol. Psychiatry.

[b27] Kohno M, Morales AM, Ghahremani DG, Hellemann G, London ED (2014). Risky decision making, prefrontal cortex, and mesocorticolimbic functional connectivity in methamphetamine dependence. JAMA Psychiatry.

[b28] Lewis CR, Staudinger K, Scheck L, Olive MF (2013). The effects of maternal separation on adult methamphetamine self-administration, extinction, reinstatement, and MeCP2 immunoreactivity in the nucleus accumbens. Front. Psychiatry.

[b29] London ED, Simon SL, Berman SM, Mandelkern MA, Lichtman AM, Bramen J (2004). Mood disturbances and regional cerebral metabolic abnormalities in recently abstinent methamphetamine abusers. Arch. Gen. Psychiatry.

[b30] van Marle HJ, Hermans EJ, Qin S, Fernandez G (2010). Enhanced resting-state connectivity of amygdala in the immediate aftermath of acute psychological stress. Neuroimage.

[b31] McCrory EJ, De Brito SA, Kelly PA, Bird G, Sebastian CL, Mechelli A (2013). Amygdala activation in maltreated children during pre-attentive emotional processing. Br. J. Psychiatry.

[b32] Meade CS, Watt MH, Sikkema KJ, Deng LX, Ranby KW, Skinner D (2012). Methamphetamine use is associated with childhood sexual abuse and HIV sexual risk behaviors among patrons of alcohol-serving venues in Cape Town, South Africa. Drug Alcohol Depend.

[b33] Messina N, Marinelli-Casey P, Hillhouse M, Rawson R, Hunter J, Ang A (2008). Childhood adverse events and methamphetamine use among men and women. J. Psychoactive Drugs Suppl.

[b34] Mingione CJ, Heffner JL, Blom TJ, Anthenelli RM (2012). Childhood adversity, serotonin transporter (5-HTTLPR) genotype, and risk for cigarette smoking and nicotine dependence in alcohol dependent adults. Drug Alcohol Depend.

[b35] Morales AM, Lee B, Hellemann G, O'Neill J, London ED (2012). Gray-matter volume in methamphetamine dependence: cigarette smoking and changes with abstinence from methamphetamine. Drug Alcohol Depend.

[b36] Nanni V, Uher R, Danese A (2012). Childhood maltreatment predicts unfavorable course of illness and treatment outcome in depression: a meta-analysis. Am. J. Psychiatry.

[b37] Neff KD (2003). The development and validation of a scale to measure self-compassion. Self Ident.

[b38] Neff KD, Germer CK (2012). A pilot study and randomized controlled trial of the mindful self-compassion program. J. Clin. Psychol.

[b39] Paivio SC, Cramer KM (2004). Factor structure and reliability of the Childhood Trauma Questionnaire in a Canadian undergraduate student sample. Child Abuse Negl.

[b40] Philip NS, Sweet LH, Tyrka AR, Price LH, Bloom RF, Carpenter LL (2013). Decreased default network connectivity is associated with early life stress in medication-free healthy adults. Eur. Neuropsychopharmacol.

[b41] Power JD, Barnes KA, Snyder AZ, Schlaggar BL, Petersen SE (2011). Spurious but systematic correlations in functional connectivity MRI networks arise from subject motion. NeuroImage.

[b42] Salo R, Flower K, Kielstein A, Leamon MH, Nordahl TE, Galloway GP (2011). Psychiatric comorbidity in methamphetamine dependence. Psychiatry Res.

[b43] Scher CD, Stein MB, Asmundson GJ, McCreary DR, Forde DR (2001). The childhood trauma questionnaire in a community sample: psychometric properties and normative data. J. Trauma. Stress.

[b44] Seggar LB, Lambert MJ, Hansen NB (2002). Assessing clinical significance: application to the beck depression inventory. Behav. Ther.

[b45] Sinha R (2001). How does stress increase risk of drug abuse and relapse?. Psychopharmacology.

[b46] Sobel MB (1982). Asymptotic confidence intervals for indirect effects in structural equation models. Sociol. Methodol.

[b47] Spielberger CD, Gorsuch RL (1983). Manual for the state-trait anxiety inventory (Form Y).

[b48] Sugaya L, Hasin DS, Olfson M, Lin KH, Grant BF, Blanco C (2012). Child physical abuse and adult mental health: a national study. J. Trauma. Stress.

[b49] Tanaka M, Wekerle C, Schmuck ML, Paglia-Boak A (2011). The linkages among childhood maltreatment, adolescent mental health, and self-compassion in child welfare adolescents. Child Abuse Negl.

[b50] Thompson PM, Hayashi K, Simon SL, Geaga JA, Hong MS, Sui Y (2004). Structural abnormalities in the brains of human subjects who use methamphetamine. J. Neurosci.

[b51] Wang Z, Du J, Sun H, Wu H, Xiao Z, Zhao M (2010). Patterns of childhood trauma and psychological distress among injecting heroin users in China. PLoS One.

[b52] Wang L, Dai Z, Peng H, Tan L, Ding Y, He Z (2014). Overlapping and segregated resting-state functional connectivity in patients with major depressive disorder with and without childhood neglect. Hum. Brain Mapp.

[b53] Weiss NH, Tull MT, Lavender J, Gratz KL (2013). Role of emotion dysregulation in the relationship between childhood abuse and probable PTSD in a sample of substance abusers. Child Abuse Negl.

[b54] van der Werff SJ, Pannekoek JN, Veer IM, van Tol MJ, Aleman A, Veltman DJ (2013). Resting-state functional connectivity in adults with childhood emotional maltreatment. Psychol. Med.

[b55] Werner KH, Jazaieri H, Goldin PR, Ziv M, Heimberg RG, Gross JJ (2012). Self-compassion and social anxiety disorder. Anxiety Stress Cop.

[b56] Witkiewitz K, Bowen S, Douglas H, Hsu SH (2012). Mindfulness-based relapse prevention for substance craving. Addict. Behav.

[b57] Woon FL, Hedges DW (2008). Hippocampal and amygdala volumes in children and adults with childhood maltreatment-related posttraumatic stress disorder: a meta-analysis. Hippocampus.

